# Exploring food consumption patterns in the province of Kenitra, Northwest of Morocco

**DOI:** 10.1186/s12889-024-19335-7

**Published:** 2024-07-16

**Authors:** Zakia Hindi, Chaimaa Belfakira, Amina Lafram, Samir Bikri, Asmaa Benayad, Hamid EL Bilali, Susanne Gjedsted Bügel, Dominika Srednicka-Tober, Patrizia Pugliese, Carola Strassner, Laura Rossi, Lilliana Stefanovic, Youssef Aboussaleh

**Affiliations:** 1 Biology and Health Laboratory, Department of Life Sciences, Faculty of Sciences, University Ibn Tofail, B.P. 133, Kenitra, 14000 Morocco; 2 International Centre for Advanced Mediterranean Agronomic Studies (CIHEAM-Bari), Valenzano (Bari), 70010 Italy; 3 Department of Nutrition, Exercise and Sports, University of Copenhagen, Frederiksberg, Denmark; 4 Department of Functional and Organic Food, Institute of Human Nutrition Sciences, Warsaw University of Life Sciences, Nowoursynowska 159c, Warsaw, 02-776 Poland; 5 Department of Food – Nutrition – Facilities, FH Münster University of Applied Sciences, Münster, Germany; 6 Council for Agricultural Research and Economics - Research Centre for Food and Nutrition, CREA – Food and Nutrition), Rome, Italy; 7 Section of Organic Food Quality, Faculty of Organic Agricultural Sciences, University of Kassel, Kassel, Germany

**Keywords:** Food consumption patterns, Mediterranean diet, Kenitra province, Morocco

## Abstract

**Background:**

Morocco is currently undergoing rapid changes in diets and lifestyles, influenced by globalization and urbanization, leading to a shift away from the Mediterranean diet (MedDiet) toward Western diets.

**Objective:**

Describe and explore the food consumption patterns of the population of Kenitra province and their adherence to the MedDiet using a validated survey.

**Method:**

The current cross-sectional study involved 442 respondents from Kenitra province, comprising individuals aged 18 and above. The survey included a combination of closed and open-ended questions regarding food consumption patterns and socio-demographic characteristics. Adherence to the MedDiet was assessed using the Panagiotakos method to calculate the MedDiet score (MDS). Additionally, each participant’s adherence to the MedDiet was evaluated using a method based on Martínez-González’s approach.

**Results:**

The results indicate that 31% of the participants eat vegetables, 28% eat fruits, and 19% eat wholegrain bread daily. Furthermore, 58% regularly use olive oil in their cooking. Eating out of home (OOH) was high, with popular places being restaurants and cafes (70%), fast food outlets (20%), and workplace canteens (7%). Overall, participants showed moderate adherence to the MedDiet, with a mean MDS of 36.3 ± 19.7.

**Conclusions:**

Promoting healthy eating habits is crucial in Kenitra. With moderate consumption of nutrient-dense foods and the popularity of the MedDiet, targeted interventions and educational initiatives can promote healthy dietary behaviors, improving overall public health.

**Supplementary Information:**

The online version contains supplementary material available at 10.1186/s12889-024-19335-7.

## Background

Eating patterns are defined as conscious, collective, and repetitive behaviors, that drive individuals to choose, consume, and incorporate certain foods or diets, in response to environmental and biological factors, social and cultural influences, such as cooking skills, taste preferences, family eating habits, and knowledge of healthy eating [1, 2].

In Morocco, there is a variety of diets followed by the population, one of them is the Mediterranean Diet (MedDiet). It is characterized by a high intake of plant-based foods (vegetables, fruits, cereals, legumes, and nuts), olive oil as the principal source of fat, moderate amounts of dairy (yogurt and cheese), low or moderate consumption of meat and fish, moderate consumption of wine, and an active lifestyle [3–5].

Like many developing countries and other MedDiet countries, Morocco is experiencing significant changes in its dietary patterns, moving away from traditional diets toward Western diets. The country is also witnessing changes in food production methods, moving from traditional processing techniques to more industrialized techniques due to urbanization, economic growth, globalization, and the food industry, and this shift has led to a noticeable nutritional transition [6, 7]. This could have a high impact on health as well as on the environment.

Western diet – that is low in vegetables, fruits, nuts, and whole grains and high in red and processed meat – is the leading contributor to the global health crisis and affects most regions [8].

Currently, hypertension, coronary heart disease (CHD), diabetes, cerebrovascular diseases, osteoarthritis, and cancer account for a significant portion of global healthcare costs [9]. Research strongly indicates that adhering to the MedDiet is linked to lower overall and cause-specific mortality rates [10, 11]. Healthy dietary practices, like the MedDiet, are associated with a lower incidence of major non-communicable diseases (NCDs) in older adults. These include cardiovascular disease (CVD) [12, 13], metabolic syndrome, obesity, and diabetes [14], some types of cancer [15], cognitive decline and dementia [16, 17], and unipolar depression [18]. Evidence also suggests a reduction in major cardiovascular risk factors such as hypertension, inflammatory markers, and dyslipidemia [19, 20]. The favorable impact of the MedDiet appear to stem from the synergistic interactions among foods and nutrients rather than their individual effects [21].

The disease pattern associated with specific dietary patterns underscores the importance of understanding and promoting healthier eating habits [22].

Fruits and vegetables are rich in various nutrients and phytochemicals such as fibers, vitamins, minerals, antioxidants, carotenoids, flavonoids and other beneficial components. These compounds act synergically through different biological mechanisms to decrease the risk of chronic diseases and premature mortality [23]. The importance of fruits and vegetables in diets is acknowledged by the World Health Organization (WHO) which emphasizes their inclusion as a way to enhance overall health and reduce the occurrence of non-communicable diseases (NCDs) [24, 25].

Furthermore, encouraging the inclusion of wholegrain products in daily meals can contribute to better overall nutrition and help individuals meet their dietary needs for essential nutrients and fiber [26].

Eating patterns are determinants of health status among households, therefore it is important to understand the factors influencing an individual’s food choices, encompassing elements like knowledge, attitudes, practices, as well as socio-cultural and economic aspects [27].

However, there are limited studies about this topic in the province of Kenitra (northwestern Morocco) [28, 29]. This paper focuses on Kenitra as a case study and aims to describe and explore the food consumption patterns of the population in this province and their adherence to the MedDiet. To clarify our investigation, we analyzed the consumption frequency of different food groups in this province including fruits and vegetables, cereals and their substitutes, meat, dairy products, fast foods and sugary drinks and many others. It is assumed that a tangible transition toward a healthier and more sustainable food system can only be achieved by reducing the consumption of industrialized refined foods and fast meals, while also maintaining the traditional Moroccan eating pattern.

## Materials and methods

This study, which is part of a project entitled Organic Agro-Food Systems as Models for Sustainable Food Systems in Europe and Northern Africa (SysOrg), was conducted in 2022 by Ibn Tofail University Faculty of Sciences (Kenitra, Morocco) in collaboration with partners from Copenhagen University (Copenhagen, Denmark), Warsaw University of Life Sciences (Warsaw, Poland), the Council for Agricultural Research and Economics (CREA; Rome, Italy), FH Münster University of Applied Sciences (Münster, Germany), University of Kassel (, Germany), and the International Centre for Advanced Mediterranean Agronomic Studies—Mediterranean Agronomic Institute of Bari (CIHEAM-Bari; Valenzano, Italy).

### Studied population

We carried out a cross-sectional study that included a total of 442 respondents from the province of Kenitra, Morocco. They were aged 18 years and above, and belonged to different socio-economic groups, educational levels and cultural backgrounds. The survey was conducted in different places in the city of Kenitra, including streets, parks, supermarkets, and universities. Data collection was either face-to-face randomly in these various locations or by telephone and online. Surveys were administered through various social media platforms, customized to suit the preferences and convenience of the participants. Individuals were excluded if they had any chronic disease or were unwilling to participate.

### Survey questionnaire

The survey was conducted from May 10th to September 11th, 2022. It consisted of a combination of closed and open-ended questions dealing with socio-demographic information, adherence to a healthy diet, nutritional knowledge, sustainable diets and food waste.

In this article, we focus on two parts: socio-demographics and adherence to a healthy diet. The first part included questions on socio-demographic characteristics: age, gender, education level, average income in Moroccan Dirhams (MAD) (The exchange rate is approximately 1 USD = 10 MAD), percentage of net household income spent on food, out-of-home eating (OOH), and primary out-of-home supplier preference. The second part of the questionnaire consisted of questions about the frequency of consumption of 24 food groups using the following frequency categories: never, less than once/month, 1–3 times/month, once/week, 2–4 times/week, 5–6 times/week, once/day, 2–3 times/day, 4–5 times/day and ″every time I eat″.

Before the main survey began, a preliminary sample of 20 respondents was recruited to test the questionnaire and ensure that the questions were clearly understood and aligned with the survey’s objectives. The translation of the survey from English into Arabic was done by an Arabic native speaker (professor at the University of Ibn Tofail).

### MedDiet score assessment

We used the Panagiotakos’s method to calculate the MedDiet score [30] which involves evaluating the frequency of consuming various food groups. These groups include non-refined cereals, fruits, vegetables, legumes, potatoes, fish, meat, poultry, full-fat dairy products, olive oil, and alcohol. Each item in the food groups was assigned a number from 0 to 5 based on its monthly consumption frequency.

This scoring system yields a total score ranging from 0 to 55, indicating adherence to the MedDiet. Higher scores reflect greater adherence.

We converted the collected food consumption frequencies using the categories provided by this method. For example, the category “never” corresponds to the “never” category in Panagiotakos’s method. Similarly, “less than once a month,” “1–3 times per month,” and “once a week” are grouped within the “1–4” per month category, and so on. In this way, we aligned our data with the consumption frequency categories used in the Panagiotakos’s method to calculate the MedDiet score.

We evaluated each participant’s adherence to the Mediterranean diet using a method based on Martínez-González’s 2012 approach [31], with some minor adjustments. The adherence scores range from 0 to 13, where 0 and 13 represent the lowest and highest adherence, respectively. A score between 5 and 9 denotes low to moderate adherence, while a score of 9 or higher signifies high adherence.

### Statistical analysis

The SPSS software for Windows (Statistical Package for the Social Sciences, version 25) was used to describe and analyze the collected data. The chi-square test evaluated the association between food group consumption and socio-demographic and socio-economic aspects. Additionally, logistic regressions were employed for the entire sample to examine potential associations between socio-economic, demographic factors and adherence to the Mediterranean diet. P-values ≤ 0.05 are considered statistically significant.

## Results

### Participant characteristics

The results show that gender was equally represented, with 50% women and 50% men. The most represented age categories were 18–34 and 35–44 years, with a frequency of 52% and 27%, respectively. About 55% of the participants had a university education, about 25% had a high school education, while 21% had no formal education or had completed only primary education. The majority of the participants were from a middle socio-economic level (43%).

### Eating out preferences: participant insights

The participants show a varied pattern of eating out, with 8.7% never dining away from home and approximately 91% engaging in out-of-home eating (OHE) at different frequencies: less than once a month (17.3%), 1–3 times per month (22.6%), once a week (20.2%), 2–4 times per week (17%), 5–6 times per week (5.4%), once a day (4.3%), 2–3 times per day (2.2%), 4–5 times per day (0.2%), and “every time I eat” (2%). Coffee shops and restaurants are the places most frequented by the studied population, comprising 70% of visits (Lmahlabat and snack bars, where traditional food is found, are considered as small restaurants by the studied population). They are followed by fast-food restaurants (19.9%), canteens at places of work (6.9%), and other places (3.2%).

Table 1 showed a noticeable disparity in the results. fruits and vegetables are consumed at least once a day by the majority of the participants, while the minority consumed them 4–5 times daily. Bread, which is the most consumed food in Morocco, varies between whole-grain and white bread consumption.

**Table 1:** Consumption frequency (%) of several food groups by the sample population of Kenitra (*n* = 442)

	The frequency of food consumption (%) by the participants (*n*)									
	Never	Less than once a month	1–3 times per month	Once a week	2–4 times per week	5–6 times per week	Once a day	2–3 times per day	4–5 times per day	Every time I eat
Fruits	1(0.2)	5(1.1)	16(3.6)	24(5.4)	98(22.2)	56(12.6)	138(31.4)	83(18.8)	1(0.2)	20(4.5)
Dried fruits	10(2.2)	43(9.6)	84(19.1)	102(23.1)	85(19.3)	20(4.5)	76(17.3)	8(1.8)	1(0.2)	13(2.9)
Vegetables (excluding potatoes)	7(1.6)	11(2.5)	9(2)	12(2.7)	81(18.4)	92(20.9)	124(28)	72(16.4)	6(1.3)	28(6.3)
Legumes	6(1.3)	27(6.1)	57(13.0)	175(39.7)	136(30.9)	10(2.2)	22(4.9)	2(0.4)	0	6(1.3)
Non-processed nuts	14(3.1)	51(11.4)	71(17.3)	120(27.4)	106(24.2)	0	51(12.3)	7(1.6)	0	12(3.1)
Whole-grain bread	17(3.8)	23(5.2)	33(7.4)	43(9.6)	52(11.9)	40(9.0)	82(18.6)	106(24.2)	12(2.7)	34(7.6)
White bread	86(19.5)	54(12.1)	49(11.0)	58(13.2)	57(13.0)	18(4.0)	67(15.2)	30(6.7)	3(0.7)	20(4.5)
Non-whole grain cereal products	43(9.6)	36(8.1)	63(14.1)	141(32.1)	118(26.9)	14(3.1)	13(2.9)	4(0.9)	0	10(2.2)
Whole grain cereal products	62(13.9)	71(16.1)	60(13.5)	121(27.6)	98(22.2)	6(1.3)	12(2.7)	3(0.7)	1(0.2)	8(1.8)
Potatoes	4(0.9)	2(0.4)	7(1.6)	17(3.8)	201(45.7)	67(15.0)	91(20.6)	36(8.1)	1(0.2)	16(3.6)
White meat	8(1.8)	9(2.0)	24(5.4)	43(9.6)	197(44.8)	38(8.5)	73(16.6)	16(3.6)	0	34(7.6)
Red meat	11(2.5)	28(6.3)	65(14.6)	134(30.5)	149(33.9)	11(2.5)	32(7.2)	3(0.7)	1(0.2)	8(1.8)
Processed meat	77(17.3)	80(18.2)	104(23.8)	89(20.2)	41(9.2)	4(0.9)	37(8.3)	1(0.2)	0	9(2.0)
Fish or shellfish	22(4.9)	41(9.2)	48(11.0)	110(24.9)	179(40.6)	9(2.0)	27(6.1)	1(0.2)	0	5(1.1)
Dairy products	6(1.3)	17(3.8)	21(4.7)	26(5.8)	98 (22.4)	73(16.4)	134 (30.5)	34(7.6)	1(0.2)	32(7.2)
Cheese	29(6.5)	38(8.5)	51(11.4)	60(13.5)	102 (23.3)	40(9.0)	96(22.0)	8(1.8)	0	18(4.0)
Butter and/or margarine	68(15.5)	64(14.6)	52(11.7)	72 (16.4)	84(19.1)	34(7.6)	57(12.8)	3(0.7)	0	8(1.8)
Eggs	13(2.9)	6(1.3)	28(6.3)	29(6.5)	178 (40.4)	52(11.7)	113(25.8)	7(1.6)	2(0.4)	14(3.1)
Alcohol	396 (89.7)	28(6.3)	9(2.0)	3(0.7)	5(1.1)	1(0.2)	0	0	0	0
Sugary drinks	50 (11.2)	43 (9.6)	48 (11.0)	40 (9.0)	94 (21.1)	31 (7.0)	90 (20.6)	34 (7.8)	1 (0.2)	11 (2.5)
Fast food	51 (11.4)	90 (20.4)	107 (24.4)	87 (19.7)	65 (14.6)	12 (2.7)	24 (5.4)	2 (2.4)	0	4 (0.9)
Desserts/sweets	36 (8.1)	42 (9.2)	62 (13.9)	100 (22.9)	95 (21.5)	13 (2.9)	79 (17.9)	6 (1.3)	1 (0.2)	8 (1.8)
Sauce	57 (12.8)	53 (11.9)	65(14.8)	102 (23.3)	98 (22.2)	13 (2.9)	40 (9.0)	5 (1.1)	3 (0.7)	6 (1.3)
Processed salty snacks	51 (11.4)	54 (12.1)	84 (19.3)	94 (21.3)	76 (17.3)	21 (4.7)	42 (9.4)	5 (1.1)	2 (0.4)	13 (2.9)

Whole-grain bread was consumed 2–3 times per day by most participants, with only a few consuming it 4–5 times daily. White bread was avoided by 86 participants. Potatoes, which are not included with vegetables, were consumed 2–4 times per week by the majority of the participants (*n* = 201). White meat, red meat, and fish were consumed 2–4 times weekly. Dairy products were consumed daily by approximately one-third of participants. Eggs were consumed 2–4 times weekly. Sugary drinks were popular, with most participants consuming them 2–4 times weekly, and fast food was consumed 1–3 times monthly by most participants.

Responses to the inquiry about dietary practices revealed significant insights. The MedDiet emerged as the most prevalent choice, embraced by 33.4% of the surveyed populace, closely followed by the flexitarian/semi-vegetarian regimen, with 17.5% adherence. Other notable diets included vegan (3.4%), lacto-ovo-vegetarian (1.3%), lacto-vegetarian (2.5%), ovo-vegetarian (1.8%), pesco-vegetarian (2.2%), lactose-free (1.3%), Paleo (2%), and gluten-free (1.6%). Notably, a substantial portion, 33%, reported not following any specific dietary regimen.

The associations between the consumption of food groups and gender, obtained using the chi-square test. As shown in this Table 2, a significant association was revealed between fruits, legumes, white bread, whole grain cereal products, red meat, cheese, eggs, fast food, and gender (*p* ≤ 0.05). In contrast, there is no significant association between gender and the other variables such as vegetables, dried fruits, non-processed nuts, whole-grain bread, non-whole grain cereal products, potatoes, white meat, fish or shellfish, dairy products and alcohol.

**Table 2:** Food consumption patterns among men and women in Kenitra (*n* = 442)

	Food groups *n* = 442	Gender		P value
		Female	Male	
Fruits	< 1 time/day	61	82	0.040
	≥ 1 time/day	159	140	
Dried fruits	< 1 time/day	160	165	0.705
	≥ 1 time/day	60	57	
Vegetables	< 1 time/day	64	55	0.310
	≥ 1 time/day	156	167	
Legumes	< 1 time/day	210	192	0.001
	≥ 1 time/day	10	30	
Non-processed nuts	< 1 time/day	186	182	0.471
	≥ 1 time/day	34	40	
Whole-grain bread	< 1 time/day	81	88	0.542
	≥ 1 time/day	139	134	
White bread	< 1 time/day	163	140	0.013
	≥ 1 time/day	57	82	
Non-whole grain cereal products	< 1 time/day	195	206	0.132
	≥ 1 time/day	25	16	
Whole grain cereal products	< 1 time/day	198	214	0.008
	≥ 1 time/day	22	8	
Potatoes	< 1 time/day	110	121	0.299
	≥ 1 time/day	110	101	
White meat	< 1 time/day	142	138	0.604
	≥ 1 time/day	78	84	
Red meat	< 1 time/day	202	185	0.007
	≥ 1 time/day	18	37	
Processed meat	< 1 time/day	199	192	0.192
	≥ 1 time/day	21	30	
Fish or shellfish	< 1 time/day	203	197	0.205
	≥ 1 time/day	17	25	
Dairy products	< 1 time/day	76	94	0.093
	≥ 1 time/day	144	128	
Cheese	< 1 time/day	122	156	0.001
	≥ 1 time/day	98	66	
Butter and/or margarine	< 1 time/day	176	164	0.127
	≥ 1 time/day	44	58	
Eggs	< 1 time/day	137	115	0.027
	≥ 1 time/day	83	107	
Alcohol	< 1 time/day	220	221	0.319
	≥ 1 time/day	0	1	
Sugary drinks	< 1 time/day	120	142	0.294
	≥ 1 time/day	90	80	
Fast food	< 1 time/day	206	194	0.025
	≥ 1 time/day	14	28	
Desserts/sweets	< 1 time/day	162	172	0.348
	≥ 1 time/day	58	50	
Sauce	< 1 time/day	192	184	0.249
	≥ 1 time/day	29	38	
Processed salty snacks	< 1 time/day	175	184	0.370
	≥ 1 time/day	45	38	

Table 3 shows the associations between diets and education level. A significant association (*p* ≤ 0.05) was revealed between legumes, non-processed nuts, white meat, red meat, fish or shellfish, processed meat, eggs, butter and or margarine, fast food, desserts/sweets, sauce, processed salty snacks and level of education. However, there is no significant association between the level of education and the other variables such as fruit, vegetables, whole-grain bread, white bread, non-whole grain cereal products, whole-grain cereal products, potatoes, dairy products, cheese, and alcohol.

**Table 3:** Determinants of dietary patterns by level of education (*n* = 442)

		Education Levels								*P* value
		No formal education	Primary education (1–4 years)	Lower secondary education (5–10 years)	Upper secondary education (10–13 years)	Apprenticeship (2–3 years)	Bachelor’s degree or equivalent level (3 years)	Master’s degree or equivalent level (e.g. Diploma) (3 + 2 years)	Doctoral studies (PhD) and/or higher	
Fruit (%)	< 1 time/day	6	15	13	28	16	35	23	9	0.348
	≥ 1 time/day	15	26	17	41	24	95	56	23	
Dried fruits	< 1 time/day	18	34	24	42	27	99	56	23	0.020
	≥ 1 time/day	3	7	6	28	13	31	22	9	
Vegetables	< 1 time/day	6	12	6	20	8	39	22	8	0.930
	≥ 1 time/day	15	29	24	50	32	91	56	24	
Legumes	< 1 time/day	21	40	28	49	36	121	77	30	< 0.001
	≥ 1 time/day	0	1	2	21	4	9	1	2	
Non-processed nuts	< 1 time/day	19	34	26	44	35	115	68	27	< 0.001
	≥ 1 time/day	2	7	4	26	5	15	10	5	
Whole-grain bread	< 1 time/day	3	19	10	21	19	57	27	13	0.096
	≥ 1 time/day	18	22	20	48	21	73	52	19	
White bread	< 1 time/day	19	25	23	39	26	95	53	24	0.061
	≥ 1 time/day	2	16	7	30	14	34	27	8	
Non-whole grain cereal products	< 1 time/day	18	40	28	65	36	112	71	31	0.270
	≥ 1 time/day	3	1	2	4	4	18	8	11	
Whole grain cereal products	< 1 time/day	18	38	30	66	34	121	74	31	0.199
	≥ 1 time/day	3	3	0	3	6	9	5	1	
Potatoes	< 1 time/day	8	19	13	28	20	76	49	18	0.060
	≥ 1 time/day	13	22	17	41	20	54	30	14	
White meat	< 1 time/day	17	24	23	30	18	93	55	20	< 0.001
	≥ 1 time/day	4	17	7	40	22	37	23	12	
Red meat	< 1 time/day	19	39	26	44	33	123	72	31	< 0.001
	≥ 1 time/day	2	2	4	26	7	7	6	1	
Processed meat	< 1 time/day	19	38	26	47	33	121	75	32	< 0.001
	≥ 1 time/day	2	3	4	23	7	9	3	0	
Fish or shellfish	< 1 time/day	21	40	26	47	39	127	71	32	< 0.001
	≥ 1 time/day	0	1	4	23	1	6	7	0	
Dairy products	< 1 time/day	8	15	15	27	14	47	32	12	0.928
	≥ 1 time/day	13	26	15	43	26	83	46	20	
Cheese	< 1 time/day	16	26	19	35	24	87	78	23	0.252
	≥ 1 time/day	5	15	11	35	16	43	30	9	
Butter and/or margarine	< 1 time/day	17	32	20	43	30	113	54	31	< 0.001
	≥ 1 time/day	4	9	10	27	10	17	24	1	
Eggs	< 1 time/day	14	24	17	27	19	83	51	19	0.021
	≥ 1 time/day	7	17	13	42	21	46	29	13	
Alcohol	< 1 time/day	21	41	30	70	40	129	78	32	0.936
	≥ 1 time/day	0	0	0	0	0	1	0	0	
Sugary drinks	< 1 time/day	14	28	21	39	22	82	47	20	0.781
	≥ 1 time/day	7	13	9	31	18	47	32	12	
Fast food	< 1 time/day	21	40	27	52	36	122	72	30	< 0.001
	≥ 1 time/day	0	1	3	18	4	8	6	2	
Desserts/sweets	< 1 time/day	20	29	25	37	25	104	69	25	< 0.001
	≥ 1 time/day	1	12	5	33	15	26	9	7	
Sauce	< 1 time/day	21	37	24	48	36	117	64	28	0.001
	≥ 1 time/day	0	4	6	22	4	13	14	4	
Processed salty snacks	< 1 time/day	19	39	24	45	28	111	65	28	0.001
	≥ 1 time/day	2	2	6	25	12	19	13	4	

The associations between the consumption of food groups and income are presented in Table 4. A significant association was revealed between fruit, dried fruits, non-processed nuts, processed meat, dairy products, cheese, desserts/sweets (*p* ≤ 0.05) and disposable net household income (*p* ≤ 0.05). In contrast, there is no significant association between disposable net household income and the other food groups such as vegetables, legumes, whole-grain bread, white bread, non-whole grain cereal products, whole grain cereal products, potatoes, white meat, red meat, fish or shellfish, butter and/or margarine, eggs, alcohol, sugary drinks, fast food, sauce, and processed salty snacks.

**Table 4:** Determinants of dietary patterns by Disposable Net Household Income (*n* = 442) in Moroccan dirhams (MAD)

		Disposable Net Household Income (MAD).							
		Up to 3.400	3.401-5.600	5.601-8.400	8.401–11.200	11.201-14.000	14.001–16.900	More than 16.900	P value
Fruit	< 1 time/day	21	45	18	7	1	1	0	0.001
	≥ 1 time/day	2	2	6	25	12	19	13	
Dried fruits	< 1 time/day	41	81	43	13	4	3	3	< 0.001
	≥ 1 time/day	8	15	6	6	4	3	4	
Vegetables	< 1 time/day	16	22	14	4	0	2	2	0.609
	≥ 1 time/day	33	74	35	15	8	4	5	
Legumes	< 1 time/day	46	89	47	19	8	6	7	0.145
	≥ 1 time/day	3	7	2	0	0	0	0	
Non-processed nuts	< 1 time/day	47	87	46	17	6	5	5	0.001
	≥ 1 time/day	2	9	3	2	2	1	2	
Whole-grain bread	< 1 time/day	19	41	16	5	0	3	4	0.254
	≥ 1 time/day	30	55	33	14	8	3	3	
White bread	< 1 time/day	31	66	40	14	7	4	4	0.443
	≥ 1 time/day	18	30	9	5	1	2	3	
Non-whole grain cereal products	< 1 time/day	45	84	47	19	7	5	6	0.597
	≥ 1 time/day	4	12	2	0	1	1	1	
Whole grain cereal products	< 1 time/day	47	93	49	19	7	6	6	0.341
	≥ 1 time/day	2	3	3	0	1	0	1	
Potatoes	< 1 time/day	22	58	33	8	4	4	4	0.127
	≥ 1 time/day	27	38	16	11	4	2	3	
White meat	< 1 time/day	27	68	37	14	4	5	6	0.060
	≥ 1 time/day	22	28	12	5	4	1	1	
Red meat	< 1 time/day	46	87	43	17	8	5	6	0.553
	≥ 1 time/day	3	9	6	2	0	1	1	
Processed meat	< 1 time/day	47	89	49	17	7	6	6	0.005
	≥ 1 time/day	2	7	0	2	1	0	1	
Fish or shellfish	< 1 time/day	47	89	48	16	7	6	7	0.131
	≥ 1 time/day	2	7	1	3	1	0	0	
Dairy products	< 1 time/day	27	50	22	10	3	4	2	< 0.001
	≥ 1 time/day	22	46	27	9	5	2	5	
Cheese	< 1 time/day	38	66	40	9	5	2	2	0.001
	≥ 1 time/day	11	30	9	10	3	4	5	
Butter and/or margarine	< 1 time/day	42	74	38	16	8	3	7	0.136
	≥ 1 time/day	7	22	11	3	0	3	0	
Eggs	< 1 time/day	27	57	36	14	2	3	5	0.062
	≥ 1 time/day	22	39	13	5	6	3	2	
Alcohol	< 1 time/day	49	96	49	19	8	6	7	0.993
	≥ 1 time/day	0	0	0	0	0	0	0	
Sugary drinks	< 1 time/day	30	67	32	14	6	2	3	0.229
	≥ 1 time/day	19	29	17	5	2	4	4	
Fast food	< 1 time/day	47	91	45	19	7	6	6	0.134
	≥ 1 time/day	2	5	4	0	1	0	1	
Desserts/sweets	< 1 time/day	35	86	42	15	6	5	2	< 0.001
	≥ 1 time/day	14	10	7	4	2	1	5	
Sauce	< 1 time/day	42	89	44	17	8	4	4	0.025
	≥ 1 time/day	7	7	5	2	0	2	3	
Processed salty snacks	< 1 time/day	42	78	43	17	8	4	6	0.400
	≥ 1 time/day	7	18	6	2	0	2	1	

All income values are reported in Moroccan Dirhams (MAD). The exchange rate is approximately 1 USD = 10 MAD

According to the results (Table 5), the majority of participants consume vegetables excluding potatoes (72.9%), fruits (67.5%), and whole-grain bread (62.1%) more than 18 times per month. Additionally, 58.4% of participants used olive oil frequently in their cooking. The participants had a moderate adherence to the MedDiet, with an overall MDS score of 36.3 ± 19.7.

**Table 5:** The MedDiet score

Food groups (%)	Never	1–4/ month	5–8/ month	9–12/ month	13–18/ month	> 18/ month	MedDiet score
Fruits	1.3	9	7.4	7.4	7.4	67.5	**36.26** ± **19.71**
Vegetables	4	4.6	6.3	6.1	6.1	72.9	
Legumes	8.2	58.1	11.3	11.3	11.1	0	
Whole-grain bread	9	17	4	4	3.9	62.1	
Other whole grain cereal	30	41	7.4	7.4	7.4	6.8	
Potatoes	1.3	5.4	15.2	15.2	15.3	47.6	
White meat	3.8	15	15	15	14.8	36.4	
Red meat	8.7	45.1	11.4	11.2	11.3	12.3	
Fish or shellfish	14.1	35.9	13.7	13.5	13.5	9.3	
Dairy products	5.2	10.5	7.4	7.6	7.4	61.9	
Cheese	15	25	7.6	7.8	7.8	36.8	
Alcohol	95.9	2.7	0.5	0.5	0.2	0.2	
Olive oil	8.1	5.7	9.7	13.3	4.8	58.4	

*N = 442

We used binary logistic regression to analyze factors affecting adherence to the MedDiet. The results are shown in Table 6, with adherence to the diet as the dependent variable. Age has a significant effect on adherence to the MedDiet, with a regression coefficient indicating a positive significant association (B = 0.03, *p* = 0.013, OR = 1.03). Gender has a coefficient of -0.5, suggesting a negative effect on adherence (*p* = 0.018, OR = 0.61). Education level (B = 0.182, *p* = 0.004, OR = 1.20) and disposable net household income (B = 0.14, *p* < 0.001, OR = 1.15) are both positively associated with adherence. However, the percentage of monthly income spent on food (B=-0.03, *p* = 0.804, OR = 0.97) and eating out of home (B = 0.03, *p* = 0.64, OR = 1.03) showed no significant association.

**Table 6:** Logistic regression analysis of factors influencing adherence to Mediterranean Diet

	B	*P*-value	OR	IC for Exp(B) 95%	
				Lower	Upper
Age (years)	0.029	0.013	1.029	1.006	1.053
Gender	-0.500	0.018	0.607	0.401	0.918
Educational level	0.182	0.004	1.200	1.061	1.357
Disposable Net Household Income	0.141	0.000	1.152	1.074	1.235
Monthly Income Spent on Food	-0.032	0.804	0.969	0.753	1.246
Eating Out of Home	0.027	0.640	1.027	0.919	1.148
Constante	-1.285	0.113	0.277		

According to the responses from residents of Kenitra province, Table 7 showed that 78.5% believe dietary experts recommend increasing fruit consumption, while 85% endorse the recommendation for vegetables and 86.1% for water intake. Moreover, 83% believe they suggest limiting foods and drinks with added sugar, and 69.5% think meat consumption should be limited.

**Table 7:** National Dietary guidelines: Health and Environment recommendations for Food groups (*n* = 442)

Food groups (%)	What do you think your National Dietary Guidelines suggest, considering health and environment, in terms of the listed food groups?			
	Increase the consumption	Limit the consumption	No specific recommendations	I am not sure
Fruits	78.5	6.5	7.8	7.2
Food and drinks with added sugar	2.2	83	8.1	6.7
Vegetables	85	4	5.2	5.8
Legumes	61.4	7.6	17.9	13
Meat	7.2	69.5	12.8	10.5
Whole grain products	66.8	7.2	12.8	13.2
Foods rich in salt	1.8	81.4	8.1	8.7
Water	86.1	6.3	2.5	5.2
Highly processed food	4.9	56.1	17.3	21.7

## Discussion

Moroccan cuisine showcases a fusion of flavors and culinary traditions from Africa, the Middle East, India, China, and Malaysia. This incorporates also elements from French, Spanish, and Portuguese cuisines, as well as customs, to shape their unique culinary heritage. It is worth noting that the customs and rituals surrounding food hold equal significance alongside the dishes themselves [32].

The present study provides insights into the food consumption patterns in Kenitra. Our results show that the frequency of daily consumption of fruits (31.4%) and vegetables (28%) is relatively low, these percentages specifically indicate the participants who consumed fruits and vegetables once a day, as shown in Table 1. This indicates a need to increase the consumption of these products in the population to improve overall dietary habits and health outcomes [33]. A significant proportion of the surveyed population demonstrates awareness of the importance of increasing fruit consumption and vegetable intake, while also recognizing the need to reduce and limit the consumption of highly processed and animal-source foods. Our findings align with a cross-sectional study conducted at the University of Dammam (Saudi Arabia), which focused on student participants [34]. The study revealed notable similarities in dietary patterns and awareness of national and international dietary guidelines. It found that the majority of participants (72%) were aware of the recommendations set forth by the World Health Organization (WHO). However, despite this awareness, the consumption of fruits and vegetables did not meet the WHO recommendations in 84% of the cases [34]. In both Morocco and Saudi Arabia there is a consensus among participants about the importance of increasing the consumption of fruits and vegetables. This aligns with the national food guidelines and recommendations set forth by international organizations such as the WHO. However, despite this awareness and acknowledgment of dietary recommendations, there are significant challenges in meeting these guidelines. Both studies indicate that a considerable portion of the population falls short of recommended intake levels for fruits and vegetables. These similarities suggest common dietary trends and challenges in both countries, highlighting the need for targeted interventions and public health initiatives to promote healthier eating habits and align dietary behaviors more closely with national and international guidelines. According to a European study [35], participants from Eastern Europe were found to have the lowest likelihood of consuming fruits and vegetables, while participants from Southern and Northern European countries had the highest likelihood of consuming fruits and vegetables [35].

In addition, the consumption of cereals, specifically wholegrain bread, appears to be the preferred choice for a majority of the population that consumes them more than 2–3 times per day (34.5%). This reveals a positive trend toward incorporating whole grains into daily meals, which can have numerous health benefits. However, the Australian Health Survey data from NNPAS 2011–2012 [26] have shown that 70% of Australians did not consume sufficient quantities of grains. Furthermore, a study conducted by Bellisle et al. [36] in France reveals that whole grain consumption is relatively low among the studied population. The participants reported consuming very small amounts of whole grains on only three to four occasions per week. It seems that French consumers enjoy bread, pasta and rice but mainly in their refined forms rather than as whole-grain products. Similarly, a study conducted in Switzerland [37] revealed that a significant number of individuals fail to consume a sufficient quantity of food containing complex carbohydrates [37]. such as whole-wheat products. However, there is a positive trend observed with whole grain versions of breakfast cereals being frequently consumed across age groups [36]. It is recommended that at least 55% of calories should come from carbohydrates [37]. Overall, these findings emphasize the importance of promoting whole grain consumption across different regions. Public health campaigns, and educational programs and increased availability of whole grain options could play a crucial role in improving grain consumption patterns and overall health outcomes in these populations.

Poultry meat consumption has increased in all world regions, mainly in North America [38]. In the present study, white meat is the preferred choice for meat consumption, followed by red meat and fish. Notably, the consumption of processed meat is lower (9.2%) with a frequency of 2–4 times per week, which indicates healthy choices in meat consumption. This trend reflects a growing awareness of the potential health risks associated with excessive intake of red and processed meats, leading individuals to opt for leaner alternatives [39]. Encouragingly, these findings highlight a conscious effort to prioritize health and make more informed dietary choices. On the other hand, a study conducted in Meknes-Tafilalet region (eastern Morocco) [40] revealed that 79% of the participants eat red and white meats 3 times per week or more, 16% eat them 1–2 times a week and 5% eat them rarely to never [40]. Another study in Casablanca city (Morocco) [41] found that the population consumes a low rate of meat.

Dairy products have a nutrient-dense profile, surpassing other typical foods in terms of calcium, protein, magnesium, potassium, zinc and phosphorus per calorie [42, 43]. Dietary guidelines often recommend three daily servings of dairy products such as a glass of milk, a portion of cheese or a yogurt [44]. Moreover, the consumption of dairy products is generally associated with minimal adverse effects [45]. Based on the results of our study, it was found that 31% of the Kenitra population consumes dairy products once a day. This aligns with findings from the National Office of Food Safety (NOFS) in Morocco [46], which indicate that the average dairy consumption in Morocco is only one serving per day, equivalent to 70 L per person per year, falling below the recommended global average of 90 L per person per year [46].

Alcohol consumption has been recognized as a contributing factor to various diseases, injuries, social problems and legal complications [47]. The 2018 report of the World Health Organization on alcohol and health [48] states that alcohol-related digestive disease fatalities are most prevalent in Africa (16.9 deaths per 100,000 people) and the Western Pacific regions (10.8 deaths per 100,000 people). Additionally, in Europe, alcohol contributes to approximately 30% of all digestive disease deaths [48]. However, when examining the specific context of Kenitra (Morocco), the research indicates a low prevalence of alcohol consumption. In this particular area, 89% of individuals reported abstaining from alcohol entirely, while only 6% admitted to consuming it once a month. These findings suggest a comparatively lower level of alcohol consumption and potentially reduced associated risks within the community.

According to our findings, there are significant associations between the consumption of fruit (*p* = 0.040), legumes (*p* = 0.001), red meat (*p* = 0.007), eggs (*p* = 0.027) and gender. However, there was no significant association found for vegetable consumption (*p* = 0.310). In comparison, the study conducted by Mohtadi et al. [41] in Casablanca revealed a significant association between vegetable consumption (*p* = 0.024), legumes (*p* = 0.035), fish (*p* = 0.001), red meat (*p* = 0.001), processed meat (*p* = 0.023), eggs (*p* = 0.022) and gender [41]. These results suggest some consistency in the observed food consumption patterns between the two cities, although some differences may also be noted, such as the significance of associations for certain food groups. Another study conducted in the Meknes-Tafilalet region of Morocco by Belloute and Diouri [40] reveals that individuals with lower educational attainment, below a high school diploma, exhibit a greater preference for meat consumption (*p* < 0.05) compared to those with higher levels of education. Conversely, individuals with higher educational backgrounds tend to favor balanced meals incorporating vegetables, legumes, fish, and cereals [40]. When comparing these findings with our results, a significant relationship emerges between dietary preferences and educational level, particularly regarding the consumption of various food groups such as legumes, white meat, red meat, fish, butter and margarine, eggs, and fast food (*p* < 0.05).

In our studied population, the MedDiet is the most popular choice, followed by 33.4% of the respondents. This way of eating emphasizes the consumption of fruits, vegetables, whole grains and healthy fats like olive oil, while limiting processed and red meat. The MedDiet has been associated with a variety of health benefits [49, 50]. A study carried out in Casablanca city (Morocco) also revealed that strong adherence to the MedDiet was marked by significant consumption of vegetables, fruits, pulses, fish, cereals, and olive oil, while meat and dairy consumption remained comparatively low, as indicated by the simplified MedDiet Score [41]. In our study, we used Panagiotakos’s method to assess adherence to the MedDiet by analyzing consumption frequencies of the same food groups included in this method. Panagiotakos’s method has been applied in various studies, such as those examining internet usage patterns’ association with the diet in South West England [51] and diet quality’s relationship with cardiovascular morbidity on Elafonisos Island, Greece [52]. Our results show that the participants had a moderate adherence to the MedDiet, with an overall MDS score of 36.3 ± 19.7. Additionally, we used the Mediterranean Diet Adherence Screener, developed by Martínez-González et al. [31]. and adapted into Turkish by Pehlivanoğlu et al. [53], to conduct binary logistic regression. Our findings revealed that Age, education, and disposable net household income positively influence adherence to the MedDiet, while gender shows a negative effect. However, spending on food and dining out did not significantly impact adherence to the MedDiet.

On the other hand, a study conducted by El Kinany et al. [54] on 1516 participants in Morocco found that 23% closely adhered to the MedDiet. A systematic review by Obeid et al. [55] examined MedDiet adherence among adults in the Mediterranean region, known for this dietary pattern. The review confirmed that, of the 50 articles assessed, most indicated moderate MedDiet adherence. Notably, 35 studies highlighted low to moderate adherence, hinting at a shift from traditional Mediterranean dietary habits in modern populations [55]. Our findings complement this trend by revealing a similar pattern of moderate adherence to the MedDiet among our study participants.

The results about OOH frequency **(91%)** of the surveyed population of Kenitra are aligned with the undergoing rapid changes in the Moroccan diet and lifestyle due to globalization and urbanization [41].

A study conducted by Barakat et al. [28] showed that **16%** of households have out-of-home breakfast at least once a week, about **50%** opted for out-of-home lunch and **11%** for out-of-home dinner. The study showed that the most visited places for OOH are fast-food restaurants **(41%)**, at least once a week, Moroccan restaurants **(35%)**, and places of work or training **(26%)**. These findings highlight the popularity of OOH in Kenitra and show diverse dining locations [28]. The present study shows that coffee shops and restaurants are the most frequented places by the studied population **(70%)**. As a growing city in recent years, Kenitra has seen the emergence of many cafes and restaurants with reasonable prices.

The growing number of eating services is closely linked to the shifts observed in the food consumption trend. OOH is a global phenomenon on the rise expanding in tandem with economic growth and the proliferation of food services and fast-food establishments [56]. The nutritional status of individuals is influenced by various factors related to OOH, including the location, frequency and food quality [57]. However, a previous study [58] associated consuming food at eateries and leisure places as well as consuming food “on the go” with making less healthy food choices and experiencing an increase in average body weight among the general population [58].

The main limitation of this study is the lack of available data regarding the quantities of foods consumed, as well as the inability to adjust for total energy intake. Additionally, there is a limitation related to the factor of disposable net household income as the majority of participants chose not to answer, potentially impacting the results regarding this variable. Nonetheless, the study possesses several strengths. It was conducted with a good sample size, participants responded to a comprehensive questionnaire, and were guided by trained interviewers to ensure accurate responses without misclassification. Overall, this study offers valuable data to advance the understanding of the association between food consumption variables and the Mediterranean diet score, in relation to socio-demographic and economic factors.

## Conclusions

The present study showed that the eating patterns of the studied population are characterized by a moderate consumption of whole, nutrient-dense foods, such as fruits, vegetables, whole grains, healthy fats like olive oil, and limited processed and red meat.

The MedDiet stands out as the most favored diet among the population of Kenitra, with a moderate prevalence of 32.5%. It is closely trailed by the flexitarian/semi-vegetarian diet, which holds a respectable proportion of 16.6%.

Drawing from the descriptive and correlational findings, it is prudent to implement initiatives aimed at promoting healthy eating habits. This could involve creating educational materials that incorporate positive eating practices, specifically targeting young individuals. And in light of the critical role of nutrition in public health, it is evident also that targeted nutritional interventions can significantly impact food choices by equipping individuals with essential information to make healthier selections, ultimately contributing to an enhanced diet. Therefore, it is crucial to comprehensively grasp the multifaceted factors influencing individual food choices, including knowledge, attitudes, practices, as well as socio-cultural and economic considerations. Understanding these elements is imperative for developing effective strategies to promote healthier dietary behaviors. Overall, the study highlights the need to promote healthy dietary habits in the population, particularly in terms of increasing the consumption of fruits, vegetables and whole grains while reducing the consumption of unhealthy food options such as fast food, sugary drinks and processed snacks.

### Electronic supplementary material

Below is the link to the electronic supplementary material.

Supplementary Material 1

## Data Availability

Data are available from the corresponding author upon reasonable request.

## Abbreviations

Sysorg: Organic agro- food systems
MedDiet: Mediterranean diet
MDS: Med Diet score
OOH: Eating out of home
WHO: World Health Organization
NCDs: Non-communicable diseases
