# Supplementary material for: Exploring food consumption patterns in the province of Kenitra, Northwest of Morocco
Source: BMC Public Health. 2024 Jul 16;24:1899. doi: 10.1186/s12889-024-19335-7 (PMC11251386; doi:10.1186/s12889-024-19335-7)
Supplement: Supplementary file 1 — Supplementary Material 1 [file 12889_2024_19335_MOESM1_ESM.docx]

**Supplementary materials**

**Table S1**. Sociodemographic characteristics of participants.

| **Characteristic** | | **N=442** | **%** |
| --- | --- | --- | --- |
| **Gender** | Women | 222 | 50 |
|  | Men | 222 | 50 |
| **Age (y)** | 18-34 | 232 | 52 |
|  | 35-44 | 116 | 27 |
|  | 44 and more | 94 | 21 |
| **Level of Education** | No formal education | 21 | 5 |
|  | Primary and middle school | 71 | 16 |
|  | High school | 108 | 24 |
|  | University | 242 | 55 |

**Table S2**. Diets followed by the studied population of Kenitra (n=442).

| Do you follow a certain diet? | |
| --- | --- |
| Vegan | 3.4 % |
| Lacto-ovo-vegetarian | 1.3 % |
| Lacto-vegetarian | 2.5 % |
| Ovo-vegetarian | 1.8 % |
| Flexitarian/semi-vegetarian diet | 17.5 % |
| Pesco-vegetarian | 2.2 % |
| Lactose free | 1.3 % |
| Gluten free | 1.6 % |
| Mediterranean | 33.4 % |
| Paleo | 2 % |
| No | 33 % |

**Figure S1.** OOH frequency (%) among the surveyed population of Kenitra (n=442).

**Figure S2.** The OOH suppliers and food services frequented by the sample population of Kenitra (n=442)

**Study questionnaire:** Sociodemographic Information, Sustainable Diet, Nutritional Knowledge, and Adherence to Healthy Eating

| **Part 1 - Sociodemographic information** | | | | | | | | | | | | | | | | | |
| --- | --- | --- | --- | --- | --- | --- | --- | --- | --- | --- | --- | --- | --- | --- | --- | --- | --- |
| **The questionnaire should be filled out by the person primarily responsible for preparing and purchasing food in your household.** | | | | | | | | | | | | | | | | | |
| 1. **Do you live in this territory [KENITRA]?** | | | | | | | | | | | | | | | | | |
| ☐Yes | | | | | | | | | | ☐No* | | | | | | | |
| 1. **Which municipality/administrative part of the city/region (district) do you live in?** | | | | | | | | | | | | | | | | | |
| Name: _____________________________ Zip Code: ________________________________ | | | | | | | | | | | | | | | | | |
| 1. **Your age (in years)** | | | | | | | | | | | | | | | | | |
|  | | | | | | | | | | | | | | | | | |
| 1. **Your gender**** | | | | | | | | | | | | | | | | | |
| ☐Female | | | ☐Male | | | | | | ☐No pronoun | | | | | ☐ I prefer not to answer | | | |
| 1. **Your level of education**** | | | | | | | | | | | | | | | | | |
| ☐No formal education | | | | | ☐Primary education (1-4 years) | | ☐ Lower secondary education (5-10 years) | | | | | | ☐  Upper secondary education (10-13 years) | | | | |
| ☐Apprenticeship (2-3 years) | | | | | ☐ Bachelor’s degree or equivalent level (3 years) | | ☐ Master’s degree or equivalent level (3+2 years) | | | | | | ☐ Doctoral studies (PhD) and/or higher | | | | |
| 1. **Household members** | | | | | | | | | | | | | | | | | |
| Total number of household members | | | | | | | | | | | | | | | | | |
| Age in Years | | | | | Female Members | | | | | | Male Members | | | | Other | | |
| < 1 | | | | |  | | | | | |  | | | |  | | |
| 1-9 | | | | |  | | | | | |  | | | |  | | |
| 10-17 | | | | |  | | | | | |  | | | |  | | |
| Adults (≥18) | | | | |  | | | | | |  | | | |  | | |
| 1. **Disposable Net Household Income (in Euro)** ** | | | | | | | | | | | | | | | | | |
| ☐Up to 36.000 | | | | | ☐36.001-60.000 | | ☐60.001-90.000 | | | | | | ☐90.001- 120.000 | | | | |
| ☐120.001 - 150.000 | | | | | ☐150.001-180.000 | | ☐More than 180.001 | | | | | | ☐I prefer not to answer | | | | |
| 1. **How much of your net monthly household income is approximately spent on food purchase per month?** | | | | | | | | | | | | | | | | | |
| ☐<10% | | | | | ☐10-30% | | ☐30-40% | | | | | | ☐>40% | | | | |
| 1. **How often do you eat food from “out-of-home” meal suppliers?** | | | | | | | | | | | | | | | | | |
| ☐Never | ☐Less than once/month | ☐1-3 times per month | | ☐Once a week | | ☐2-4 times per week | | ☐5-6 times per week | | | | ☐Once a day | ☐2-3 times per day | | | ☐4-5 times per day | ☐Every time I eat |
| 1. **What kind of “out-of-home” supplier, do you mainly use?** | | | | | | | | | | | | | | | | | |
| ☐Restaurants/cafés, including delivery and take-away) | | | | | ☐Canteen (e.g. work, nursery home, hospitals, schools) | | ☐Fast Food restaurants, including delivery and take-away | | | | | | ☐Other: ________________________ | | | | |

| **Part 2 - Adherence to Healthy Diet**** | | | | | | | | | | | | | | | | | | | | | | | | | | | | |
| --- | --- | --- | --- | --- | --- | --- | --- | --- | --- | --- | --- | --- | --- | --- | --- | --- | --- | --- | --- | --- | --- | --- | --- | --- | --- | --- | --- | --- |
| 1. **How often do you eat fruits (excluding dried fruits)?** (*Tick one box*) | | | | | | | | | | | | | | | | | | | | | | | | | | | | |
| ☐Never | ☐ Less than once a month | | ☐1-3 times per month | | | | ☐Once a week | | | ☐2-4 times per week | | | ☐5-6 times per week | | | ☐Once a day | | | ☐2-3 times per day | | | | | ☐4-5 times per day | | | ☐Every time I eat | |
| 1. **How often do you eat dried fruits?** (*Tick one box*) | | | | | | | | | | | | | | | | | | | | | | | | | | | | |
| ☐Never | ☐ Less than once a month | | ☐1-3 times per month | | | | ☐Once a week | | | ☐2-4 times per week | | | ☐5-6 times per week | | | ☐Once a day | | | ☐2-3 times per day | | | | | ☐4-5 times per day | | | ☐Every time I eat | |
| 1. **How often do you eat vegetables (excluding potatoes)?** (*Tick one box*) | | | | | | | | | | | | | | | | | | | | | | | | | | | | |
| ☐Never | ☐ Less than once a month | | ☐1-3 times per month | | | | ☐Once a week | | | ☐2-4 times per week | | | ☐5-6 times per week | | | ☐Once a day | | | ☐2-3 times per day | | | | | ☐4-5 times per day | | | ☐Every time I eat | |
| 1. *How often do you eat legumes (e.g. beans, peas, lentils)? (Tick one box*) | | | | | | | | | | | | | | | | | | | | | | | | | | | | |
| ☐Never | ☐ Less than once a month | | ☐1-3 times per month | | | | ☐Once a week | | | ☐2-4 times per week | | | ☐5-6 times per week | | | ☐Once a day | | | ☐2-3 times per day | | | | | ☐4-5 times per day | | | ☐Every time I eat | |
| 1. **How often do you eat non-processed nuts, including peanuts (e.g. unsalted, non-roasted, not sugar-coated)?** *(Tick one box)* | | | | | | | | | | | | | | | | | | | | | | | | | | | | |
| ☐Never | ☐ Less than once a month | | ☐1-3 times per month | | | | ☐Once a week | | | ☐2-4 times per week | | | ☐5-6 times per week | | | ☐Once a day | | | ☐2-3 times per day | | | | | ☐4-5 times per day | | | ☐Every time I eat | |
| 1. **How often do you eat whole-grain bread?** (*Tick one box*) | | | | | | | | | | | | | | | | | | | | | | | | | | | | |
| ☐Never | ☐ Less than once a month | | ☐1-3 times per month | | | | ☐Once a week | | | ☐2-4 times per week | | | ☐5-6 times per week | | | ☐Once a day | | | ☐2-3 times per day | | | | | ☐4-5 times per day | | | ☐Every time I eat | |
| 1. **How often do you eat white bread?** (*Tick one box*) | | | | | | | | | | | | | | | | | | | | | | | | | | | | |
| ☐Never | ☐ Less than once a month | | ☐1-3 times per month | | | | ☐Once a week | | | ☐2-4 times per week | | | ☐5-6 times per week | | | ☐Once a day | | | ☐2-3 times per day | | | | | ☐4-5 times per day | | | ☐Every time I eat | |
| 1. **How often do you eat non-whole grain cereal products (e.g. Pasta, rice)??** (*Tick one box*) | | | | | | | | | | | | | | | | | | | | | | | | | | | | |
| ☐Never | ☐ Less than once a month | | ☐1-3 times per month | | | | ☐Once a week | | | ☐2-4 times per week | | | ☐5-6 times per week | | | ☐Once a day | | | ☐2-3 times per day | | | | | ☐4-5 times per day | | | ☐Every time I eat | |
| 1. **How often do you eat whole grain cereal products (e.g. pasta, rice)?** (*Tick one box*) | | | | | | | | | | | | | | | | | | | | | | | | | | | | |
| ☐Never | ☐ Less than once a month | | ☐1-3 times per month | | | | ☐Once a week | | | ☐2-4 times per week | | | ☐5-6 times per week | | | ☐Once a day | | | ☐2-3 times per day | | | | | ☐4-5 times per day | | | ☐Every time I eat | |
| 1. **How often do you eat potatoes?** (*Tick one box*) | | | | | | | | | | | | | | | | | | | | | | | | | | | | |
| ☐Never | | ☐ Less than once a month | | | ☐1-3 times per month | | | | ☐Once a week | | | ☐2-4 times per week | | | ☐5-6 times per week | | | ☐Once a day | | | ☐2-3 times per day | | | | | ☐4-5 times per day | | ☐Every time I eat |
| 1. **How often do you eat white meat (e.g. rabbit, chicken, turkey, other poultry)?** (*Tick one box*) | | | | | | | | | | | | | | | | | | | | | | | | | | | | |
| ☐Never | ☐ Less than once a month | | ☐1-3 times per month | | | | ☐Once a week | | | ☐2-4 times per week | | | ☐5-6 times per week | | | ☐Once a day | | | ☐2-3 times per day | | | | | ☐4-5 times per day | | | ☐Every time I eat | |
| 1. **How often do you eat red meat (e.g. beef, pork, veal, lamb)?** (*Tick one box*) | | | | | | | | | | | | | | | | | | | | | | | | | | | | |
| ☐Never | ☐ Less than once a month | | ☐1-3 times per month | | | | ☐Once a week | | | ☐2-4 times per week | | | ☐5-6 times per week | | | ☐Once a day | | | ☐2-3 times per day | | | | | ☐4-5 times per day | | | ☐Every time I eat | |
| 1. **How often do you eat processed meat (e.g. cured ham and turkey, salami)? (Tick one box)** | | | | | | | | | | | | | | | | | | | | | | | | | | | | |
| ☐Never | ☐ Less than once a month | | ☐1-3 times per month | | | | ☐Once a week | | | ☐2-4 times per week | | | ☐5-6 times per week | | | ☐Once a day | | | ☐2-3 times per day | | | | | ☐4-5 times per day | | | ☐Every time I eat | |
| 1. **How often do you eat fish or shellfish, including processed fish (e.g. canned tuna, smoked salmon)?** (*Tick one box*) | | | | | | | | | | | | | | | | | | | | | | | | | | | | |
| ☐Never | ☐ Less than once a month | | ☐1-3 times per month | | | | ☐Once a week | | | ☐2-4 times per week | | | ☐5-6 times per week | | | ☐Once a day | | | ☐2-3 times per day | | | | | ☐4-5 times per day | | | ☐Every time I eat | |
| 1. **How often do you eat/drink dairy products (excluding cheese) (e.g. milk, yogurt, buttermilk, kefir, skyr and other fermented dairy products)? (Tick one box)** | | | | | | | | | | | | | | | | | | | | | | | | | | | | |
| ☐Never | ☐ Less than once a month | | ☐1-3 times per month | | | | ☐Once a week | | | ☐2-4 times per week | | | ☐5-6 times per week | | | ☐Once a day | | | ☐2-3 times per day | | | | | ☐4-5 times per day | | | ☐Every time I eat | |
| 1. **What kind of milk/plant-based drink do you mainly drink?** *(Tick one box)* | | | | | | | | | | | | | | | | | | | | | | | | | | | | |
| ☐Whole fat cow milk | | | | | ☐ Semi-skimmed cow milk | | | | | | | ☐Skimmed cow milk | | | | | | ☐Plant based milk (e.g., oat, pea, almond, soy, coconut) | | | | | | | | ☐None of these | | |
| 1. **How often do you eat cheese?** (*Tick one box*) | | | | | | | | | | | | | | | | | | | | | | | | | | | | |
| ☐Never | ☐ Less than once a month | | ☐1-3 times per month | | | | ☐Once a week | | | ☐2-4 times per week | | | ☐5-6 times per week | | | ☐Once a day | | | ☐2-3 times per day | | | | | ☐4-5 times per day | | | ☐Every time I eat | |
| 1. **How often do you eat butter and/or margarine?** (*Tick one box*) | | | | | | | | | | | | | | | | | | | | | | | | | | | | |
| ☐Never | ☐ Less than once a month | | ☐1-3 times per month | | | | ☐Once a week | | | ☐2-4 times per week | | | ☐5-6 times per week | | | ☐Once a day | | | ☐2-3 times per day | | | | | ☐4-5 times per day | | | ☐Every time I eat | |
| 1. **How often do you eat eggs?** (*Tick one box*) | | | | | | | | | | | | | | | | | | | | | | | | | | | | |
| ☐Never | ☐ Less than once a month | | ☐1-3 times per month | | | | ☐Once a week | | | ☐2-4 times per week | | | ☐5-6 times per week | | | ☐Once a day | | | ☐2-3 times per day | | | | | ☐4-5 times per day | | | ☐Every time I eat | |
| 1. **How often do you drink alcohol (e.g., wine, beer)?** (*Tick one box*) | | | | | | | | | | | | | | | | | | | | | | | | | | | | |
| ☐Never | ☐ Less than once a month | | ☐1-3 times per month | | | | ☐Once a week | | | ☐2-4 times per week | | | ☐5-6 times per week | | | ☐Once a day | | | ☐2-3 times per day | | | | | ☐4-5 times per day | | | ☐Every time I eat | |
| 1. **What kind of alcohol do you mainly drink?** *(Tick one box)* | | | | | | | | | | | | | | | | | | | | | | | | | | | | |
| ☐Wine | | | | | | ☐Beer | | | | | | | | | ☐Spirits (e.g. vodka, gin, rum and similar) | | | | | | | | ☐Other: | | | | | |
| 1. **How often do you drink sugary drinks (e.g. coke, orange soda), including fruit juices?** (*Tick one box*) | | | | | | | | | | | | | | | | | | | | | | | | | | | | |
| ☐Never | ☐ Less than once a month | | ☐1-3 times per month | | | | ☐Once a week | | | ☐2-4 times per week | | | ☐5-6 times per week | | | ☐Once a day | | | ☐2-3 times per day | | | | | ☐4-5 times per day | | | ☐Every time I eat | |
| 1. **How often do you eat fast food (e.g. burgers, shawarma, hot dog or similar)?** (*Tick one box*) | | | | | | | | | | | | | | | | | | | | | | | | | | | | |
| ☐Never | ☐ Less than once a month | | ☐1-3 times per month | | | | ☐Once a week | | | ☒2-4 times per week | | | ☐5-6 times per week | | | ☐Once a day | | | ☐2-3 times per day | | | | | ☐4-5 times per day | | | ☐Every time I eat | |
| 1. **How often do you eat desserts/sweets (e.g. ice cream, cake)?** (Tick one box) | | | | | | | | | | | | | | | | | | | | | | | | | | | | |
| ☐Never | | ☐ Less than once a month | | ☐1-3 times per month | | | | ☐Once a week | | | ☐2-4 times per week | | | ☐5-6 times per week | | | ☐Once a day | | | ☐2-3 times per day | | | | | ☐4-5 times per day | | ☐Every time I eat | |
| 1. **How often do you eat** sauce (incl. hot and cold sauces, e.g., béchamel, béarnaise sauce and ketchup, mayonnaise, cocktail sauce) (*Tick one box*) | | | | | | | | | | | | | | | | | | | | | | | | | | | | |
| ☐Never | | ☐ Less than once a month | | ☐1-3 times per month | | | | ☐Once a week | | | ☐2-4 times per week | | | ☐5-6 times per week | | | ☐Once a day | | | ☐2-3 times per day | | | | | ☐4-5 times per day | | ☐Every time I eat | |
| 1. **How often do you eat processed salty snacks (e.g. nuts, crisps, popcorn, crackers, pretzels)?** (*Tick one box*) | | | | | | | | | | | | | | | | | | | | | | | | | | | | |
| ☐Never | | ☐ Less than once a month | | ☐1-3 times per month | | | | ☐Once a week | | | ☐2-4 times per week | | | ☐5-6 times per week | | | ☐Once a day | | | ☐2-3 times per day | | | | | ☐4-5 times per day | | ☐Every time I eat | |
| 1. **What kind of oil/fat do you mainly use to cook and flavour?** *(Tick maximum 3 boxes)* | | | | | | | | | | | | | | | | | | | | | | | | | | | | |
| ☐Olive oil | | | | | | | ☐Butter | | | | | | | ☐Rapeseed oil | | | | | | | | ☐Sunflower oil | | | | | | |
| ☐Ghee | | | | | | | ☐Corn oil | | | | | | | ☐Flaxseed oil | | | | | | | | ☐Sesame oil | | | | | | |
| ☐Coconut oil | | | | | | | ☐Vegan butter | | | | | | | ☐Other: ___________________________________________ | | | | | | | | | | | | | | |

| **Part 3 - Nutritional Knowledge** | | | | |
| --- | --- | --- | --- | --- |
| **This is not a test to evaluate your knowledge but a survey. Your answers will help us to identify which Dietary Guidelines are not clear yet. If you do not know how to answer, fill in the questionnaire with ‘I’m not sure’ option rather than try to guess.** | | | | |
| 1. **What kind of Dietary Guidelines do you think dietary experts, considering health and environment, suggest in terms of the listed food groups** *(Tick one box for each item)* | | | | |
|  | Increase the consumption | Limit the consumption | No specific recommendations | I am not sure |
| Fruits | ☐ | ☐ | ☐ | ☐ |
| Food and drinks with added sugar | ☐ | ☐ | ☐ | ☐ |
| Vegetables | ☐ | ☐ | ☐ | ☐ |
| Legumes |  |  |  |  |
| Meat (incl. red meat) | ☐ | ☐ | ☐ | ☐ |
| Whole grain products | ☐ | ☐ | ☐ | ☐ |
| Foods rich in salt | ☐ | ☐ | ☐ | ☐ |
| Water | ☐ | ☐ | ☐ | ☐ |
| Highly processed food | ☐ | ☐ | ☐ | ☐ |

| **Part 4 - Sustainable Diet** | | | | |
| --- | --- | --- | --- | --- |
| 1. **Do you follow a certain diet?** | | | | |
| ☐ Vegan  (Do not eat any animal products or by-products) | ☐ Lacto-ovo vegetarian  (Do eat dairy products and egg products. Do not eat red or white meat, fish or fowl) | ☐ Lacto vegetarian  (Do eat dairy products, e.g., cheese, milk and yoghurt. Do not eat red or white meat, fish, fowl or eggs) | ☐ Ovo vegetarian  (Do eat egg products. Do not eat red or white meat, fish, fowl or dairy products) | ☐ Flexitarian/semi-vegetarian diet  (Eats primarily a vegetarian diet and eats occasionally fish and/or meat) |
| ☐ Pesco-vegetarian (Do not eat red meat or poultry, but eats fish and shellfish) | ☐ Lactose free  (Do not eat milk and milk products) | ☐Gluten free  (Do not eat food that contains the gluten, including wheat, rye and barley) | ☐Mediterranean  (Do eat whole grains, fruits, vegetables, beans, herbs, spices, nuts and healthy fats such as olive oil as core food) | ☐ Paleo  (Do not eat processed foods, grains and sugar) |
| ☐ No | ☐ Other |  |  |  |
